# Honey as an antioxidant therapy to reduce cognitive ageing

**DOI:** 10.22038/IJBMS.2019.14027

**Published:** 2019-12

**Authors:** Khairunnuur Fairuz Azman, Rahimah Zakaria

**Affiliations:** 1 Department of Physiology, School of Medical Sciences, Universiti Sains Malaysia, 16150 Kubang Kerian, Kota Bharu, Kelantan, Malaysia

**Keywords:** Ageing, Antioxidant, Cognition, Honey, Oxidative stress

## Abstract

This paper reviews the potential role of honey as a therapeutic antioxidant to reduce oxidative stress and improve cognitive ageing. All articles indexed to PubMed Central (PMC) were searched using the following key words: honey, antioxidant, memory and ageing. Honey is a natural insect-derived product with therapeutic, medicinal and nutritional values. Antioxidant properties of honey quench biologically-circulating reactive oxygen species (ROS) and counter oxidative stress while restoring the cellular antioxidant defense system. Antioxidant properties of honey may complement its nootropic effects to reduce cognitive ageing.

## Introduction

Ageing is associated with a slow deterioration of cognitive functions, especially learning and memory (1, 2). Increasing age is a significant risk factor for dementia, Alzheimer’s disease and other prevalent neurodegenerative disorders (3). The accumulation of oxidative damage and the reduction of the antioxidant defense system play key roles in an organism’s ageing and functional senescence. The central nervous system is particularly susceptible to oxidative stress due to its high oxygen consumption and its lower levels of scavenging enzymes for ROS as well as endogenous antioxidants compared with other organs (4). Based on previous studies, increased protein oxidation and lipid peroxidation in various regions of aged mammalian brains have been reported (5-7). These findings suggested that oxidative damage which is increased in age-related cognitive functions could not be prevented by natural antioxidant defense mechanisms and also stated that dietary intake of antioxidants might be beneficial for preserving brain function.

Honey is a therapeutic antioxidant which contains varying concentrations of flavonoids, phenolic acids, glucose oxidase, catalase, carotenoid derivatives, ascorbic acid, organic acids, Maillard reaction products, amino acids and proteins (8-16). It also contains choline and acetylcholine, which are essential for brain functions as they act as neurotransmitters (17). Hence, recent studies on the therapeutic properties of honey have revealed that honey consumption improved cognitive function in adult rats (18), middle-aged rats (19), aged rats (20, 21), ovariectomized rats (22) and postmenopausal women (23). It was hypothesized that honey’s cognitive-enhancing effects are mainly mediated by its antioxidant properties (20-22, 24). This review aims to assess the capability of honey as a therapeutic antioxidant to protect against oxidative damage in ageing brain and improve cognition.

**Cognitive changes in the ageing brain**

The brain undergoes pronounced age-related structural and physiological changes that might influence the functional changes. Age-related structural changes can be detected at the macroscopic level (e.g., prefrontal volume loss, grey matter atrophy, changes in white matter frontostriatal systems, decline in white matter integrity, parietal cortical thinning and hippocampal damage) (25-31), the cellular level (e.g., mitochondrial changes, synaptic pruning, axonal loss and alterations to glial cell numbers) (32-34) and the molecular level (e.g., disrupted calcium signaling, altered gene expression and epigenetic changes) (32, 35, 36).

Firstly, age-related prefrontal volume loss has been shown to result in the deterioration of executive functions (37). Similarly, frontal areas and the striatum undergo significant structural changes with age (30). Buckner (38) suggested that the relatively mild memory problems often seen in physiological ageing are caused by grey matter atrophy and by changes in the white matter frontostriatal systems, in addition to the changes in the corresponding neurotransmitter systems. Other studies, on the other hand, have reported that the integrity of white matter declines with age (26, 28, 29, 31, 39) and that this deterioration is accompanied by a cognitive decline (26, 39, 40). Besides, the thinning of the parietal cortex in the elderly was also associated with episodic memory-retrieval problems (27, 38, 41).

The hippocampus, an extension of the temporal part of the cerebral cortex, plays an important role in the memory of both humans and rodents (42-44). Damage to the rat hippocampus leads to non-spatial task impairments (25), including object recognition memory (45-46), transitive odor associations (47), temporal order memory (48, 49) and social transmission of food preferences (50, 51). In young animals, the hippocampus receives highly processed multi-modal information from widespread cortical association areas (52). The hippocampus receives sensory information largely through the perforant path, which projects from the entorhinal cortex (EC) to the dentate gyrus (DG), through the CA3 and CA1 areas, to the subiculum (53). Moreover, Scheff *et al.* (54) reported a reduced number of synapses receiving EC input in the outer molecular layer of the DG in elderly subjects with mild cognitive impairment compared to cognitively-intact elderly subjects. Where the extent of synapse reduction correlated with memory ability. Similarly, aged memory-impaired humans exhibited marked atrophy and synapse loss in the perforant path (55).

On the other hand, the hippocampal neurons also undergo age-related changes, such as decreased synaptic plasticity, decreased neuronal excitability, slower neurogenesis and faster potentiation decay (2, 56-59). Adult neurogenesis is present in the aged brain, but it is dramatically reduced in early adulthood in both the subventricular zone (60, 61) and the subgranular layer of the DG (62-66). This age-related decline in hippocampal neurogenesis, together with other cellular changes, contributes to a physiological decline in cognitive functions (67, 68).

**Oxidative stress, antioxidants and the ageing brain**

*Oxidative Stress*

The brain is particularly susceptible to oxidative damage due to its rich abundance of polyunsaturated fatty acids and other lipids, its high-energy demands and high oxygen consumption rate, and its limited antioxidant capacity compared to other organs (69). Oxidative damage has been found to be the highest in the brain regions that are responsible for the cognitive dysfunctions found in Alzheimer’s disease (70-72). Oxidative stress might cause neuron dysfunction through a cascade of molecular events: (i) oxidative damage in mitochondria leads to additional free radical production, (ii) increased oxidation leads to increased levels of β-amyloid (Aβ), which generates additional amyloid precursor protein (APP), (iii) Aβ and the accumulating oxidative damage activate caspases, which cleave APP and generate additional Aβ and oxidative damage, and (iv) progressive oxidative damage and increasing Aβ concentrations cause a decrease in BCL-2 levels, leaving neurons more vulnerable to oxidative damages and insults (73). 

Oxidative damage may also affect replication and transcription of mitochondrial DNA (mtDNA) which leads to a decline in mitochondrial function, consecutively leading to enhanced ROS production and further damage to mtDNA (74). Evidently, age-dependent increases in DNA oxidative damage have also been demonstrated in the brain of Alzheimer’s disease patients (69, 75-77). Apart from humans, the accumulation of DNA oxidative damage in different tissues including the brain during ageing has also been observed in other mammals like rodents (78-83). 

The long-term oxidative stress in brain tissues possibly contributes to declining cognitive processes (84). Based on a previous study, aged hippocampal neurons which appear to be under oxidative stress is more severe in the learning-impaired subjects, suggesting a possible basis for age-induced cognitive decline (85). Indeed, increased oxidative damage is related to reductions in learning (7, 86-88), memory (87, 89, 90), and increased anxiety (91, 92). All these findings suggested that oxidative stress contributes to learning and memory deficits due to brain oxidative damages during ageing. Hence, protection from oxidative damage offers a basic approach to the controlled strategy of age-related cognitive impairment.

*Antioxidants*

ROS are highly reactive molecules that consist of a number of diverse chemical species including superoxide anion (O_2_^•−^), hydroxyl radical (•OH), and hydrogen peroxide (H_2_O_2_). ROS readily attack DNA and generate a variety of DNA lesions, such as oxidized DNA bases, abasic sites and DNA strand breaks, which ultimately lead to genomic instability. Due to their potentiality to causing oxidative damage, ROS have been implicated as one of the mechanisms underlying cognitive ageing (94).

In order to protect cells from oxidative damage, aerobic metabolism generally depends on stringent control of ROS by antioxidants. Antioxidants are substances that, when present at low concentrations than that of the oxidizable substrates, significantly inhibits or delays oxidative processes of that substrate by oxidizing itself (95). In tissues functioning normally, a balance is maintained between ROS generation and antioxidant protection, which is mediated through antioxidant enzymes and antioxidant molecules that are obtained naturally through nutrition (84). Oxidative stress leading to lipids, proteins, ribonucleic acid (RNA) and DNA oxidative damage occurs when the balance between free radical generation and antioxidant capacities shift toward free radical generation (69). 

There are two types of antioxidants in the human body, enzymatic and non-enzymatic (96). Enzymatic antioxidants, which are also known as natural antioxidants, neutralize excessive ROS and prevent them from damaging the cellular structure. Enzymatic antioxidants include superoxide dismutase (SOD), glutathione peroxidase (GPx), ascorbate peroxidase, mono- and dehydroascorbate reductases, glutathione reductase (GR), and catalase (CAT). The levels of enzymatic antioxidants tend to decrease with age, leaving the biological system vulnerable to oxidative stress, thus, contributing to the senescence processes. Tiana *et al.* (97) reported that SOD, GPx, and CAT activities in most tissues including brain, liver, heart, and kidney significantly decreased during ageing. The continuous decline of these enzymatic enzymes is associated with an increase in brain oxidative stress and age-related decline in cognitive performance (98). In addition to enzymatic antioxidants, non-enzymatic antioxidants (synthetic antioxidants) also serve as an essential biological defense against environmental pro-oxidant conditions. The complex antioxidant system of the body is influenced by the dietary intake of antioxidant vitamins and minerals, such as vitamin C, vitamin E, glutathione, melatonin, selenium, zinc, taurine, beta carotene, and carotene (99). 

Additionally, several studies have found possible inverse associations between high plasma levels of antioxidants and cognitive decline (100-103) as well as dementia (104-106). Hence, the consumption of antioxidant compounds was found to improve cognitive performance in aged animals (73, 107, 108) as well as in humans (109, 110). Research by Fahnestock *et al.* (111) revealed an increase in brain-derived neurotrophic factor (BDNF) levels, which is one of the factors underlying improvements in learning and memory in aged dogs receiving antioxidant-fortified diet. Overall, these results suggested that an increase in the intake of natural antioxidants may help maintain an acceptable antioxidant status, perhaps normal physiological functioning including cognitive function (112).

**Honey, its constituents and medicinal effects**

Honey is a naturally sweet substance. It is produced by honey bees from the nectar of plants or from secretion of living parts of plants or excretion of plant-sucking insects on the living parts of plants, which the bees collect, transform by combining with specific substances of their own, deposit, dehydrate, store, and leave in honeycombs to ripen and mature (113). The color of the honey usually ranging from water white to dark amber, depends on the floral source and its mineral content collected by the bees. Moreover, the flavor of the honey depends upon the color, normally the darker the honey the stronger the flavor and quality. Honey is often named according to the geographical location where it was produced, the floral source of the honey or the trees on which the hives are found (114). 

In general, honey can be grouped into either monofloral or multi-floral types (115). Monofloral honey is made primarily from the nectar of one type of flower, whereas multi-floral honey, also known as polyfloral or wildflower honey, is derived from the nectar of many types of flowers (115, 116). Examples of monofloral honey are manuka honey, sourwood honey, tupelo honey, orange blossom honey, acacia honey and palmetto honey, whereas examples of polyfloral honey are tualang honey, Georgia wildflower honey, savannah honey and Charleston honey. 

A number of factors such as geographical origin, botanical sources of nectar, environmental, and climatic conditions as well as the processing techniques can influence the composition of honey (117, 118). Approximately 79.6% of honey is of sugars and the major sugars are comprised of levulose and dextrose which constitutes 38.2% and 31.3% correspondingly, while the remaining 1.3% is sucrose and 7.3% is maltose (119). The minor constituents of honey include 0.57% acids, 0.27% protein, 0.04% nitrogen, 0.1% amino acids, 0.17% minerals, and other compounds such as pigments, flavor and aroma substances, phenolic compounds, colloids, sugar alcohols, and vitamins which all together account for the 2.1% of whole honey composition (119). Vitamins such as vitamin A, C, and E are also found in honey (119-121). Besides, honey also contains other bioactive constituents such as flavonoids, carotenoid-derived compounds, nitric oxide metabolites, and Maillard reaction products (118, 123-125). The primary flavonoid compounds found in honey are triacetin, quercetin, and luteolin (126). Honey also contains a number of enzymes such as glucose oxidase, diastase, invertase, phosphatase, catalase, and peroxidase (124, 127).

The use of honey in folk medicine dates back to 2100-2000 BC (128). Initially, most of the health benefits ascribed to honey were based on mere observations or generalizations without any scientific evidence. In the last few years, however, there has been a renewed interest in research related to potential health benefits of honey in the treatment of various diseases resulting in findings attributable to several medicinal effects of honey. These include cardioprotective (129), hepatoprotective (131, 132), hypoglycemia (133, 134), reproductive (139, 140), and antihypertensive effects (121, 142). Other effects such as antibacterial (142-144), antifungal (145, 146), antiviral (147), anti-inflammatory (148-150), and antitumor (119, 151-155) have also been documented and attributed to honey.

Malaysian tualang honey is produced by the rock bee (*Apis dorsata*). The rock bees build hives on tualang trees (*Koompassia excelsa*) found mainly in the north-western region of Peninsular Malaysia. Tualang honey has a dark brown appearance with a pH of 3.55–4.00 and a specific gravity of 1.335 (156). Hydrocarbons constitute more than half (58.5%) of its composition. These include alcohols, ketones, aldehydes, furans, terpenes, flavonoids, and phenols (157). A total of six phenolic acids (gallic, syringic, benzoic, trans-cinnamic, p-coumaric, and caffeic acids) and five flavonoids (catechin, kaempferol, naringenin, luteolin, and apigenin) were found in tualang honey (157-159). It was reported that tualang honey contains more phenolic acids and flavonoids than manuka honey and other local Malaysian honey (159). It was also reported that tualang honey possessed the highest antioxidant activity compared to gelam honey, Indian forest honey, and pineapple honey (159). In addition, Moniruzzaman *et al.* (160) reported that among four different types of local honey (tualang, acacia, pineapple, and Borneo), tualang honey possessed the highest phenolic compounds, flavonoid contents, ferric reducing power values along with the greatest color intensity, indicating its antioxidant potential. Tualang honey has been reported to possess medicinal properties such as anticancer (153), antiproliferative (161), anti-inflammatory, and antibacterial properties (142, 162). It has also been reported to improve male (122, 162-164) and female reproductive systems (139, 165, 167).

**Antioxidant and cognitive enhancing properties of honey**

The therapeutic role of honey in the treatment of various diseases has been attributed to its antioxidant (168, 169), anti-inflammatory and anti-acetylcholinesterase (AChE) properties (Table 1). Honey contains both enzymatic (catalase, glucose oxidase, peroxidase) and non-enzymatic antioxidants (ascorbic acid, α-tocopherol, carotenoids, amino acids, proteins, flavonoids, and phenolic acids) (179-181). The amount and type of these antioxidants depend largely upon the types of honey. Some reports have established a correlation between floral origins and phenolic compounds and flavonoids (179, 182-184). Correspondingly, a correlation between total phenolic content in honey and its antioxidant activity has been demonstrated (169,180). Other studies have also demonstrated that honey is able to increase plasma antioxidants and ameliorate oxidative stress in tissues (121, 133, 134, 137, 138, 149, 185-188).

These antioxidant properties of honey may help in reducing oxidative damage and improving brain cell structure and integrity, hence enhancing cognitive performance. Al-Himyari (24) reported that one tablespoon of honey daily to mild cognitive impaired subjects aged 65 and older was able to prevent cognitive decline and development of dementia. In addition, supplementation of a combination of astragalus honey, sedge, and saffron in major neurocognitive disorder patients was able to improve their cognitive scores, as assessed by Addenbrooke’s Cognitive Scale (189). However, another study which was conducted in rats reported that reduction in anxiety (190) and improvement in spatial memory among honey-fed middle-aged rats compared to those sucrose-fed or on a sugar-free diet (19). 

**Table 1:** Summary of therapeutic properties of honey related to its neurological effects

**Properties**	**Actions**	**Reference(s)**
**Antioxidant**	Tualang honey supplementation (20 mg/day) for 16 weeks was able to reduce blood oxidative stress levels/activities and improve memory function of postmenopausal women compared to those who received estrogen-progestin therapy	(170)
	Daily consumption of tualang honey (200 mg/kg/day) for 18 days in female ovariectomized rats is able to reduce blood oxidative stress levels/activities in the brain homogenate and improve memory performance of tualang honey-treated group as compared with the untreated stressed ovariectomized rats	(171)
	Both short and long term (short term - 3 weeks and long term - 12 weeks) honey supplementations at a dose of 250 mg/kg body weight increased the brain protein and CAT activities of brain cells	(172)
	Tualang honey supplementation was able to improve memory performance and decrease oxidative stress levels in rats exposed to noise stress	(20, 21)
	Treatment with tualang honey and its methanolic fraction markedly reduced oxidative damage in rats’ hippocampus as evidence by restoration in the activities of antioxidant enzymes (CAT, GPx and GPr) and decreased in MDA level comparable to LPS rats treated with memantine (a controlled drug)	(173)
	Tualang honey has therapeutic potential against KA-induced oxidative stress and neurodegeneration through its antioxidant effect	(174)
**Anti-inflammatory**	Tualang honey and its methanolic fraction significantly reduced the concentration of COX-2 and TNF-α expression, as well as amyloid deposition in the hippocampus of LPS rats comparable to memantine group	(175)
	Tualang honey reduced neuroinflammation and caspase-3 activity after kainic acid-induced status epilepticus	(176)
**Anti AChE**	Reduced memory performance, low concentrations of ACh and high AChE in the brain homogenates of stressed ovariectomized rats compared with non-stressed sham-operated controls and the effects were reversed after treatment with Tualang honey	(177)
	Tualang honey supplementation was able to improve memory performance and decrease acetylcholinesterase activity in noise-induced male rats	(20)
**BDNF**	BDNF concentration is significantly decreased in stressed ovariectomized rats compared to other experimental groups where the concentration was restored to normal following tualang honey treatment	(178)
	Tualang honey supplementation was able to improve memory performance, increase BDNF concentration in noise-induced male rats	(20)

Tualang honey, in particular, has been shown to improve immediate memory of postmenopausal women, a result comparable with the memory improvement seen in women receiving estrogen plus progestin therapy (23). Additionally, tualang honey also exhibited neuroprotective potential in neurodegenerative disorders as it protects the viability of hippocampal cells in chronic cerebral hypoperfusion induced by permanent bilateral common carotid arteries ligation in rats (191). Similarly, tualang honey improved short- and long-term memory of ovariectomized rats via enhancing neuronal proliferation in hippocampal CA2, CA3, and DG regions (22). Tualang honey has also been shown to improve memory performance in aged male rats, while its antioxidant properties are thought to be one of the possible mechanism (20, 21). These reports suggest the potential use of honey as an alternative therapy to prevent memory decline due to oxidative stress exposure and/or ageing in humans.

Apart from its cognitive enhancing properties in aged subjects (rats), honey has also shown similar effects in young or adult subjects. Azman *et al*. (18) reported that tualang honey could improve working memory performance in rats exposed to loud noise stress, as assessed by the novel object recognition test. Tualang honey has also been shown to improve spatial memory performance in adult male rats as assessed by the radial arm maze, via enhancing numbers of CA1 and CA3 hippocampal pyramidal cells (192). Additionally, honey exhibited neuroprotective effects against lead-induced cognitive deficit via enhancing antioxidant activities as evidenced by increased brain SOD, GST, and GSH activities (193). Another study demonstrated that honey effectively inhibited apoptosis or neuronal cell death in the hippocampus of streptozotocin-induced diabetic rats (194).

Also, at old age, the antioxidant defense systems may adopt less efficiently to defend against ROS generation compared to young age (195, 196). Azman *et al.* (21) reported that the neuroprotective effects of tualang honey were more prominent in young than old rats. While tualang honey was able to improve memory performance in both young and aged rats, it was noted the young rats exhibited lower brain MDA level and higher antioxidant enzymes activities compared to the aged rats (21). This finding is probably due to the optimal antioxidant protection system in younger groups which efficiently reduces oxidative stress. Therefore, for old subjects, longer duration or a higher dose of honey is probably needed to produce the same results as in young subjects.

Based on the results from these previous studies, positive correlations between neuronal cell counts, antioxidant activities, and memory performance were unravelled (20-22, 192). These studies confirmed the notion that exposure to oxidative stress in the course of ageing leads to damages of neuronal cells and neuronal death which eventually results in cognitive decline. Hence, antioxidant properties of honey may help combat against damages to the neuronal cells by neutralizing the free radicals, by maintaining healthy neuronal cells to preserve cognitive functions. 

## Conclusion

Oxidative stress plays an important role in ageing and neurodegenerative disorders. Based on the *in vitro *and *in vivo* studies discussed in this review, honey has the potential to be a therapeutic agent against oxidative damage and cognitive decline associated with ageing. Honey supplementation is likely to enhance the antioxidant defense system via attenuating ROS attacks on cell structures, particularly brain cells, hence preserving brain functions and cognitive ability. 

## Conflicts of Interest

The authors declare that there is no conflict of interest regarding the publication of this paper. 
